# The genome of *Ricinus communis* encodes a single glycolate oxidase with different functions in photosynthetic and heterotrophic organs

**DOI:** 10.1007/s00425-020-03504-0

**Published:** 2020-11-10

**Authors:** Jessica Schmitz, Meike Hüdig, Dieter Meier, Nicole Linka, Veronica G. Maurino

**Affiliations:** 1 Plant Molecular Physiology and Biotechnology Division, Institute of Developmental and Molecular Biology of Plants, Heinrich Heine University, and Cluster of Excellence on Plant Sciences (CEPLAS), Düsseldorf, Germany; 2 Molecular Plant Physiology Division, Institute of Molecular Physiology and Biotechnology of Plants, University of Bonn, Kirschallee 1, 53115 Bonn, Germany; 3 Institute for Plant Biochemistry, Heinrich Heine University, and Cluster of Excellence on Plant Sciences (CEPLAS), Düsseldorf, Germany

**Keywords:** 2-Hydroxy acid oxidase, Folate metabolism, Glycolate, Glyoxylate, Photorespiration, Serine biosynthesis

## Abstract

**Main conclusion:**

The biochemical characterization of glycolate oxidase in *Ricinus communis* hints to different physiological functions of the enzyme depending on the organ in which it is active.

**Abstract:**

Enzymatic activities of the photorespiratory pathway are not restricted to green tissues but are present also in heterotrophic organs. High glycolate oxidase (GOX) activity was detected in the endosperm of *Ricinus communis*. Phylogenetic analysis of the Ricinus l-2-hydroxy acid oxidase (Rc(l)-2-HAOX) family indicated that Rc(l)-2-HAOX1 to Rc(l)-2-HAOX3 cluster with the group containing streptophyte long-chain 2-hydroxy acid oxidases, whereas Rc(l)-2-HAOX4 clusters with the group containing streptophyte GOX. Rc(l)-2-HAOX4 is the closest relative to the photorespiratory GOX genes of Arabidopsis. We obtained Rc(l)-2-HAOX4 as a recombinant protein and analyze its kinetic properties in comparison to the Arabidopsis photorespiratory GOX. We also analyzed the expression of all Rc(l)-2-HAOXs and conducted metabolite profiling of different Ricinus organs. Phylogenetic analysis indicates that Rc(l)-2-HAOX4 is the only GOX encoded in the Ricinus genome (RcGOX). RcGOX has properties resembling those of the photorespiratory GOX of Arabidopsis. We found that glycolate, the substrate of GOX, is highly abundant in non-green tissues, such as roots, embryo of germinating seeds and dry seeds. We propose that RcGOX fulfills different physiological functions depending on the organ in which it is active. In autotrophic organs it oxidizes glycolate into glyoxylate as part of the photorespiratory pathway. In fast growing heterotrophic organs, it is most probably involved in the production of serine to feed the folate pathway for special demands of those tissues.

**Electronic supplementary material:**

The online version of this article (10.1007/s00425-020-03504-0) contains supplementary material, which is available to authorized users.

## Introduction

Glycolate is a metabolic intermediate produced in high amounts during photorespiration in all oxygenic photosynthetic organisms (Zelitch et al. 2009; Maurino and Peterhansel 2010). As part of the photorespiratory pathway, land plants and Charophyta convert glycolate to glyoxylate through glycolate oxidase (GOX) (Leliaert et al. 2012; Esser et al. 2014).

GOX belongs to the l-2-hydroxy acid oxidase ((l)-2-HAOX) family (Esser et al. 2014). The *Arabidopsis thaliana* genome encodes three GOXs that oxidize glycolate and l-lactate using oxygen as the electron acceptor (Engqvist et al. 2015). GOX1 and GOX2 are mostly involved in photorespiration, while GOX3 supports l-lactate metabolism in roots (Engqvist et al. 2015; Dellero et al. 2016). Animals also possess GOX (Kohler et al. 1999; Ryan et al. 2001), which has a common eukaryotic ancestor with plant GOX (Esser et al. 2014). Animal GOX produces glyoxylate, which is then used for the peroxisomal synthesis of glycine (Williams et al. 2000). Animal and plant GOX convergently duplicated to evolve long-chain 2-hydroxy acid oxidases (lHAOX), which have a broad substrate specificity (Jones et al. 2000; Esser et al. 2014). In mammals, lHAOX play a role in the degradation of food components or xenobiotic compounds (Belmouden and Lederer 1996; Verhoeven et al. 1997, 1998). Plant lHAOX are likely involved in the conversion or degradation of 2-hydroxy acids produced during the metabolism of fatty acids or amino acids (Esser et al. 2014). Arabidopsis possesses two lHAOX, which prefer long-chain fatty acids and short-chain hydroxy acids such as l-lactate, leucic acid, valic acid, and isoleucic acid over glycolate as substrates (Esser et al. 2014).

In plants several enzymes associated with the photorespiratory pathway are present in roots and other heterotrophic tissues (Lernmark et al. 1991; Igamberdiev et al. 1997). For example, in the scutellum of maize, glycine decarboxylase is involved in the oxidation of glycine formed from glyoxylate (Igamberdiev et al. 1997). Glyoxylate, the product of the GOX reaction, was shown to be metabolized to glycine and serine by different plant heterotrophic organs, such as germinating cotyledons, roots, coleoptiles, and storage tissue (Sinha and Cossins 1965). Previous work indicated the existence of GOX activity in germinating castor bean (*Ricinus communis*) endosperm (Tanner and Beevers 1965), which was also related to the synthesis of glycine (Cossins and Sinha 1967). Thus, as in the case of animals, in plant heterotrophic organs GOX may be involved in metabolic processes other than photorespiration (Maurino and Engqvist 2015).

Here, we aimed the identification and biochemical characterisation of the protein responsible for the GOX activity of heterotrophic organs of Ricinus. We found that Rc(l)-2-HAOX4 is the protein with GOX characteristics and is encoded in the Ricinus genome by a single copy gene. We cloned Rc(l)-2-HAOX4 from Ricinus endosperm and found that it has properties similar to Arabidopsis photorespiratory GOX. We propose that in heterotrophic organs, RcGOX is likely involved in the metabolism of glycolate to produce serine to feed the folate pathway for special demands of those tissues.

## Materials and methods

### Sequence alignment and phylogenetic analysis

Rc(l)-2-HAOX1 (EEF33207.1), Rc(l)-2-HAOX2 (EEF33202.1), Rc(l)-2-HAOX3 (EEF33208.1) and Rc(l)-2-HAOX4 (EEF42631.1) sequences were retrieved from NCBI using *Arabidopsis thaliana* and *Homo sapiens* GOX as queries in blastp searches. Sequences were aligned with previously characterized GOX sequences from higher plants and Animalia according to Esser et al. (2014). 21 sequences were aligned with MAFFT using Guidance2, performing 400 bootstrap iterations and an alignment score threshold for non-reliable columns of 0.8 (Katoh et al. 2002; Sela et al. 2015). Evolutionary analyses were conducted in MEGA7 (Kumar et al. 2016). The evolutionary gene tree was inferred using the Maximum Likelihood method with the Le Gascuel 2008 model (Le and Gascuel 2008). A discrete Gamma distribution was used to model evolutionary rate differences among sites [5 categories (+ *G*, parameter = 1.3901)].

### Plant growth and sample collection

Seeds of *Ricinus communis var. zanzibariensis* (Samen Aders GmbH & Co.KG, Düsseldorf, Germany) were surface sterilized with a 1% (v/v) sodium hypochlorite solution for 5 min and subsequently imbibed for 48 h in running water. Imbibed seeds were transferred to boxes of watered Vermiculite and incubated 4 days at 30 °C in darkness. Germinated seeds were either harvested and separated into germinated embryo and endosperm or transferred to vermiculite pots for further growth under a 12/12 h light/dark period and 23/20 °C cycle. The light intensity was around 90–100 µmol photons·m^−1^ s^−2^ from Spectralux Plus NL 36 W/840 (Radium) light bulbs. Plants were fertilized on a weekly basis with N/P/K fertilizer (Bayer). Leaf and root material was harvested after 5 weeks in the light. Three to four individual samples were pooled for one biological replicate, and all plant material was immediately shock frozen in liquid nitrogen.

### RNA extraction, reverse transcription and quantitative real-time PCR

Total RNA was isolated from grinded plant material according to Logemann et al. (1987). Quality and quantity of total RNA was verified in gel and photospectrometrically, respectively. Genomic DNA contaminations were removed using Ambion DNA-free DNA Removal Kit. 1.5 µg of total RNA were used for reverse transcription into complementary DNA (cDNA) using RevertAid Reverse Transcriptase (Thermo Scientific) and OligodT primer according to the manufacturer’s instruction. KAPA SYBR FAST qPCR Master Mix (Kapabiosystems) was used for real-time PCR in an Applied Biosystems Step one Plus real time PCR system with 2 µl of 1:10 diluted cDNA according to the manufacturer’s instructions. The reference gene elongation factor 1b (EF1b), with the most stable expression pattern in seed development, and primer pair (EF1b_F + EF1b_R; Table S1) were taken from Cagliari et al. (2010) and compared to a second reference gene, ubiquitin conjugating enzyme E2 (UBC, XM_002509871.3, UBC_F + UBC_R; Table S1), for which the Arabidopsis homolog (UBC9, At4g27960) has been shown to be the most stably expressed gene in developmental series. Primers for Rc(L)-2-HAOX genes (Table S1) were designed using Primer-Blast (Ye et al. 2012), and relative transcript abundance in particular tissue types was analysed from three biological samples and expressed as fold change in delta C_T_.

### Heterologous expression and purification of RcHAOX4

Complementary DNA (cDNA) of Ricinus endosperm was used as a template for PCR with the primer pair Rc4-pET_F + Rc4-pET_R (Table S1), which introduces a BamHI restriction site at the 5′ and 3′ termini of the full-length coding sequence. The PCR products were sub-cloned into pCR-Blunt-II-TOPO (Life Technologies, Invitrogen) resulting in pTOPO-Rc(l)-2-HAOX4 and confirmed via sequencing. The obtained Rc(l)-2-HAOX4 sequence revealed the presence of a valine instead of the isoleucine at position 216 expected from the sequence retrieved from NCBI. As valine at position 216 is conserved in Arabidopsis, maize, and spinach GOX proteins, the substitution is not expected to affect the protein function and most likely represents the actual GOX sequence in *Ricinus communis var. zanzibariensis.* The plasmid pTOPO-Rc(l)-2-HAOX4 was digested with BamHI and the purified DNA fragment was ligated into the BamHI linearized pET16b.

The expression vector pET16b-HIS:Rc(l)-2-HAOX4 was transformed into *E. coli* BL21 Rosetta pLysS strain (Novagen). Protein expression and Histidine-tag (HIS) based purification was performed as described in Schmitz et al. (2017a). Protein purity was verified via SDS-PAGE according to Laemmli (1970). Protein concentration was determined using the amidoblack precipitation protocol of (Schaffner and Weissmann 1973).

### Determination of enzymatic parameters of Rc(l)-2-HAOX4

Enzymatic activity was determined as described in Schmitz et al. (2017b) by measuring the change in absorption at 324 nm by the formation of phenylhydrazone using a Synergy HT Plate reader (Biotek). The standard assay mixture contained 0.5 mM EDTA, 5 mM MgSO_4_, 3 mM phenylhydrazin, 2 mM FMN, and 5 mM of either glycolate, l-lactate, or d-lactate. The assay mixture was buffered with 100 mM MES-NaOH to work at pH 5.5–6.5 or with TRIS–HCl to work at pH 7.0–9.0. Substrate affinity (*K*_m_) was deduced from saturation kinetics using nonlinear regression, fitting the Michaelis–Menten equation [*f*(*x*) = *a*·*x*/(*b* + *x*)] to the combined data from three independent protein purifications. Arabidopsis GOX1 (AtGOX1) (Engqvist et al. 2015) was assayed in parallel as a positive control.

### Cloning, transient expression, and subcellular localization of RcHAOX4

Rc(l)-2-HAOX4 was cloned as a C-terminal and N-terminal fusion protein into the vector peqFP611 (Forner and Binder 2007) containing the red fluorescent protein FP611. To generate the N-terminal fusion, two inserts were generated: the primers Rc4_N-term_fusion_F and Rc4_N-term_fusion_R (Table S1) were used to amplify Rc(l)-2-HAOX4 coding sequence from pTOPO-Rc(l)-2-HAOX4 and the primers FP611_F and FP611_R (Table S1) were used to generate a stop codon free FP611 coding sequence from peqFP611. The vector peqFP611 was digested with the restriction enzymes XbaI and SmaI. These two inserts and the backbone were combined via Gibson assembly (Gibson et al. 2009) and the resulting pFP611:RcHAOX4 was verified by sequencing. The primers Rc4_C-term_fusion_F and Rc4_C-term_fusion_R were used to amplify Rc(l)-2-HAOX4 coding sequence from pTOPO-Rc(l)-2-HAOX4. The vector peqFP611 was digested using XhoI and SacI and both fragments combined as above. The resulting pRc(l)-2-HAOX4:FP611 was verified by sequencing. Both pFP611:Rc(l)-2-HAOX4 and pRc(l)-2-HAOX4:FP611 were transformed into the *Agrobacterium tumefaciens* strain GV3101 (pMP90) and cultivated for transient transformation of *Nicotiana benthamiana* plants. Tobacco plants were grown in green house conditions during 4 weeks. Infiltration and protoplastation of tobacco leaves was conducted after 2 and 3 days of transient protein expression according to Waardt and Kudla (2008). As Rc(l)-2-HAOX4 is strongly predicted to be localized to the peroxisomes, *N*-bodipy ((8-(4-Nitrophenyl) bodipy, Biozol) was used as an organelle marker for peroxisomes (Landrum et al. 2010). Staining was achieved by adding 5 µM *N*-bodipy to the isolated protoplast 10 min before imaging.

Imaging was performed at the LSM 780 confocal microscope (Zeiss) with acquisition settings as described in Schmitz et al. (2017a) and *N*-bodipy (excitation: 488 nm, emission: 503–530 nm) fluorescence detected in a separate frame. False color images were processed using the Fiji software with ImageJ version 1.53c (Schindelin et al. 2012).

### Metabolite analysis

Total metabolites were extracted from ground material of seeds, embryo of germinated seeds, endosperm, leaf, and root according to Fiehn (2007) and Weckwerth et al. (2004) in a methanol/chloroform/water mix (5:2:2, by vol.). Ribitol was used for internal standardization. In brief a dried fraction of the extraction mixture was derivatisized with methoxyamine hydrochloride at 37 °C for 90 min and subsequently with *N*-methyl-*N*-(trimethylsilyl)trifluoroacetamide (MSTFA) at 37 °C for 30 min. Extracts were analysed by GC/MS using an accurate mass Q-TOF GC/MS system with a HP-5MS column with 5% phenyl methyl siloxane film (Agilent 7890A GC system, Agilent Technologies). A metabolite standard mixture including all metabolite targets was measured every 30 samples as retention and response reference. Raw data was processed with MassLynx and QuanLynx software (Waters GmbH) to identify metabolites in comparison to the NIST14 Mass Spectral Library (https://www.nist.gov/srd/nist-standard-reference-database-1a-v14). Response peak areas were integrated and were normalized to the internal ribitol standard and to gram of dry weight as a measure of relative metabolite content. Relative metabolite levels of three biological replicates were averaged. For comparison across different tissue types, the highest abundance of each particular metabolite was set to 1.

## Results

### The genome of Ricinus encodes four l-2-hydroxy acid oxidases

**Fig. 1:**
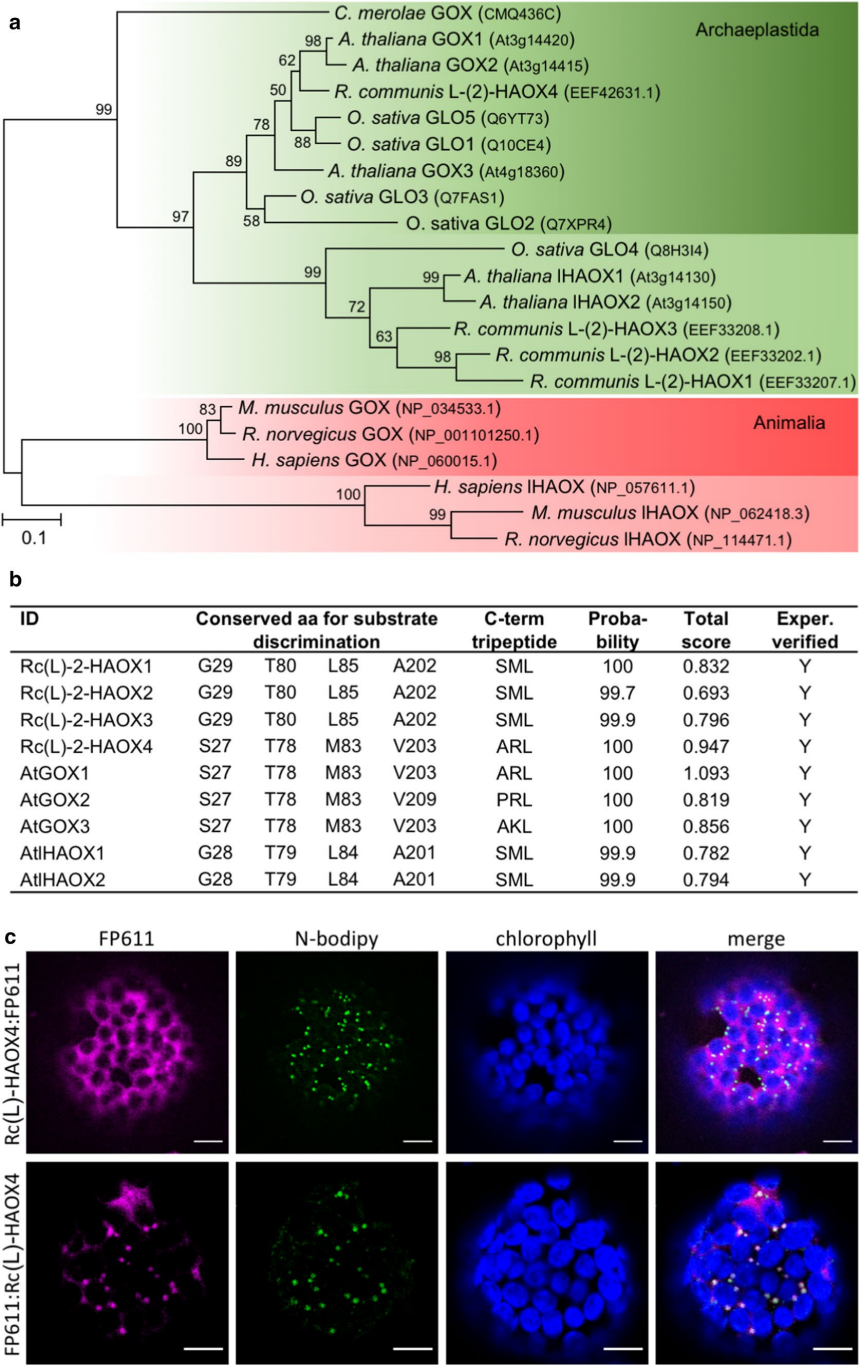
Phylogenetic and sequence analysis of (l)-2-HAOXs proteins. **a** Selected (L)-2-HAOXs sequences (Esser et al. 2014) were aligned with MAFFT using Guidance2 and the alignment filtered for unreliably aligned positions was used for subsequent analysis (Katoh et al. 2002; Sela et al. 2015). The evolutionary history was inferred using the Maximum Likelihood method based on the Le_Gascuel_2008 model (Le and Gascuel 2008). A discrete Gamma distribution was used to model evolutionary rate differences among sites (5 categories (+ *G*, parameter = 1.3901)). The tree with the highest log likelihood (-6484.48) is shown. The percentage of trees in which the associated taxa clustered together is shown above the branches (1000 Bootstraps). The tree is drawn to scale, with branch lengths proportional to the number of substitutions per site (scale bar). The analysis involved a reduced set of 21 l-(2)-HAOX amino acid sequences (gene identifier in brackets; Esser et al. 2014). There were in total 355 positions in the final dataset. **b** Conserved amino acid positions that allow a discrimination of (l)-2-HAOX into lHAOX and GOX and their different predicted substrate specificities, and sequence of the terminal tripeptide and the probability of peroxisomal targeting via a PTS1 signal, of (l)-2-HAOXs of Ricinus and Arabidopsis. The targeting prediction was performed with PredPlantPTS1 (https://ppp.gobics.de/, Threshold score 0.412, max score Arabidopsis 1.188) (Reumann et al. 2012). Exper. verified means that the PTS1 signal [SML (Lingner et al. 2011), ARL (Lingner et al. 2011), AKL (Lingner et al. 2011), PRL (Reumann et al. 2007)] has already been verified experimentally as a functional plant PTS1 tripeptide for peroxisomal targeting. **c** False color visualization of fluorescence in isolated *N. benthamiana* protoplasts. C-terminal fusion of FP611 to Rc(l)-2-HAOX4 (FP611:Rc(l)-2-HAOX4, magenta) results in cytosolic localization (top row). N-terminal fusion (Rc(l)-2-HAOX4:FP611, magenta, bottom row) colocalizes with *N*-bodipy fluorescence (green) in peroxisomes. Chlorophyll fluorescence depicted as blue. Scale bar = 10 µm

GOX and lHAOX in plants and animals most likely arose from an ancestral eukaryotic peroxisomal (l)-2-HAOX (Esser et al. 2014). A phylogenetic analysis using sequences of Ricinus (l)-2-HAOX and experimentally confirmed lHAOX and GOX proteins separate Animalia and Plantae sequences into two clades (Fig. 1a). Animalia and Streptophyta sequences each split further into GOX and lHAOX groups (Fig. 1a). This points to the convergent diversifications of the (l)-2-HAOX gene family into GOX and lHAOX subfamilies in these two eukaryotic kingdoms (Esser et al. 2014). From the four Ricinus (l)-2-HAOX proteins, Rc(l)-2-HAOX1 to Rc(l)-2-HAOX3 cluster with the group containing streptophyte lHAOX, whereas Rc(l)-2-HAOX4 clusters with the group containing streptophyte GOX.

Apart from the phylogenetic relationships, our previous work showed that four amino acid positions allow the discrimination of (l)-2-HAOX with different predicted substrate specificities (Esser et al. 2014). The analysis of these positions further confirmed that Rc(l)-2-HAOX1 to -3 possess the conserved amino acids of lHAOX sequences, while Rc(l)-2-HAOX4 possesses the conserved amino acids of GOX sequences (Fig. 1b). Moreover, computational prediction of the subcellular localization indicate that all examined sequences contain a peroxisomal targeting signal type 1 PTS1) sequence at the C-terminus (Fig. 1b; Reumann et al. 2012). All PTS1 signals [SML (Lingner et al. 2011), ARL (Lingner et al. 2011), AKL (Lingner et al. 2011), PRL (Reumann et al. 2007)] have been verified experimentally as a functional plant PTS1 tripeptide before (Fig. 1b).

To support the predicted subcellular localization, we cloned and transiently expressed fluorescent fusion proteins of Rc(l)-2-HAOX4 in tobacco leaves (Fig. 1c). We found that a C-terminal fusion protein (Rc(l)-2-HAOX4:FP611, Fig. 1c top row) localizes to the cytosol and an N-terminal fusion protein (FP611:Rc(l)-2-HAOX4, Fig. 1c, bottom row), leaving the C-terminal PTS1 of Rc(l)-2-HAOX4 free, colocalizes with an established peroxisomal marker in tobacco protoplasts. Taken together, these findings support the localization and physiological role of Rc(l)-2-HAOXs in peroxisomes.

### *Rc(l)-2-HAOX4*, the ortholog of Arabidopsis photorespiratory GOX, is highly expressed in heterotrophic organs

The Arabidopsis genome encodes three GOX isoforms; photorespiratory AtGOX1 and AtGOX2 are highly expressed in photosynthetic organs, while AtGOX3 is expressed in roots, where it supports l-lactate oxidation (Engqvist et al. 2015). Analysis of the unambiguous phylogenetic clustering and the conservation of amino acids determining substrate specificity point to the existence of a single GOX and three (l)-2-HAOX proteins in Ricinus (Fig. 1a).

**Fig. 2:**
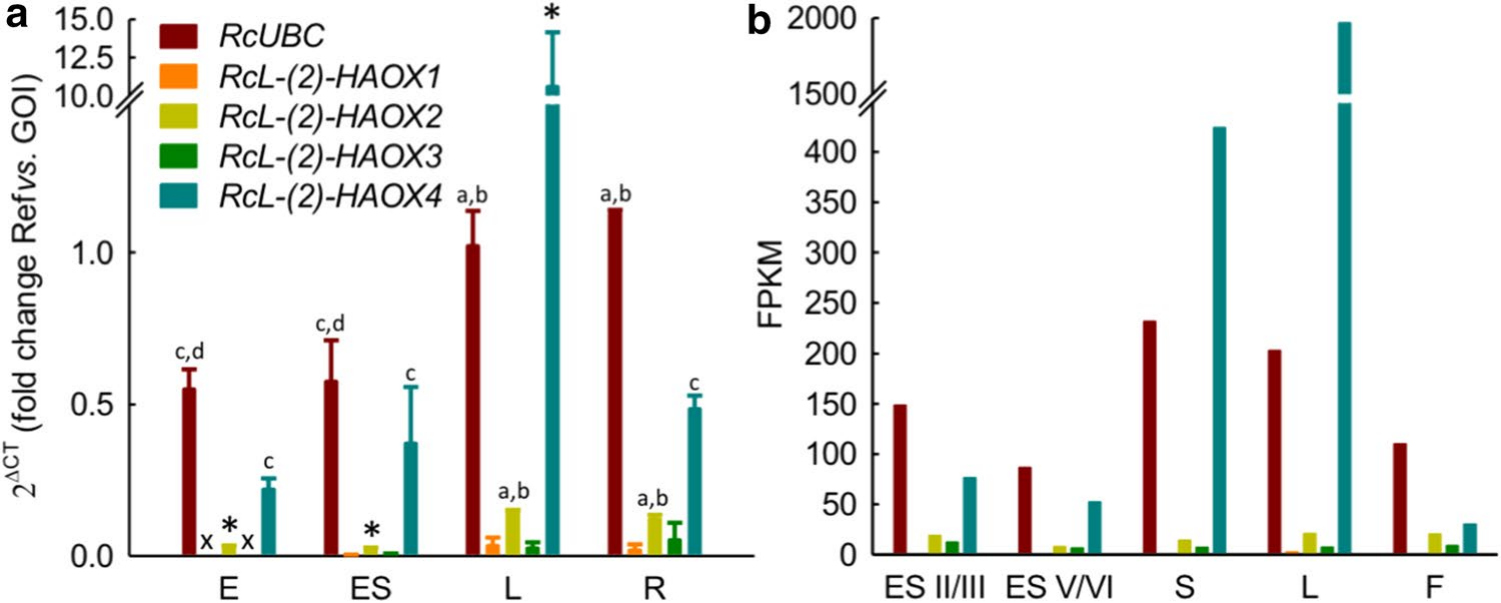
Transcript analyses of Ricinus *(l)-2-HAOXs* in heterotrophic and autotrophic tissues. **a** Quantitative real-time PCR data expressed in fold change of Δ*C*_T_, visualising expression strength across different organs. *RcUBC* expression is shown as a reference. *n* = 3, ± SE; *x* not determined, *E* embryo of germinated seeds, *ES* endosperm of germinated seeds, *L* leaf, *R* root. All genes of interest were normalized to the housekeeping gene *EF1b* and tested for significant differences with one-way Anova with subsequent Tukey post-hoc test. * Indicate significant difference to all others: *a* = significant difference to *E*, *b* = significant difference to ES, *c* = significant difference to *L*, *d* = significant difference to *R*. **b** *Rc(l)-2-HAOX1* to *Rc(l)-2-HAOX4* transcript abundances in fragments per kilobase of transcript per million mapped reads (FPKM) compared to the housekeeping gene *RcUBC* (Brown et al. 2012). ES II/III = endosperm of developing seeds at stage II/III, ES V/VI = endosperm of developing beans at stage V/VI. *S* whole germinating seed, *L* leaf, *F* flower. Stages of developing beans were taken from (Greenwood and Bewley 1982)

To gain knowledge on the abundance of *(l)-2-HAOX* mRNA in Ricinus, we performed transcriptional analysis via qPCR using heterotrophic and autotrophic organs. The expression strength of all *l-(2)-HAOX* was compared to Ubiquitin (*UBC*) (Fig. 2a). *Rc(l)-2-HAOX1 and Rc(l)-2-HAOX3* are expressed at very low levels in roots and leaves, and expression is negligible in embryos and the endosperm of germinated seeds. The expression levels of *Rc(l)-2-HAOX2* is low in all organs analysed and is comparable to that of *Rc(l)-2-HAOX1 and Rc(l)-2-HAOX3* in roots and leaves. *Rc(l)-2-HAOX4* is highly expressed in leaves. Intriguingly, we also found expression of *Rc(l)-2-HAOX4* in roots, embryos, and the endosperm of germinated seeds, with levels similar to *UBC*.

We broadened the study by analyzing available deep-sequencing RNAseq data derived from different Ricinus autotrophic and heterotrophic organs (Fig. 2b) (Brown et al. 2012). In line with the qPCR results, the expression of *Rc(l)-2-HAOX1* to *Rc(l)-2-HAOX3* is very low (FPKM < 20, i.e., less than 20 fragments per kilobase million) in all organs analysed. *Rc(l)-2-HAOX1* transcripts are only detected in leaves, while *Rc(l)-2-HAOX2* and *Rc(l)-2-HAOX3* transcripts are found at comparable low levels in all organs analysed. *Rc(l)-2-HAOX4* expression is very high in leaves (FPKM = 1966), followed by whole germinating seeds (FPKM = 424).

### Rc(l)-2-HAOX4 is a glycolate oxidase

**Fig. 3:**
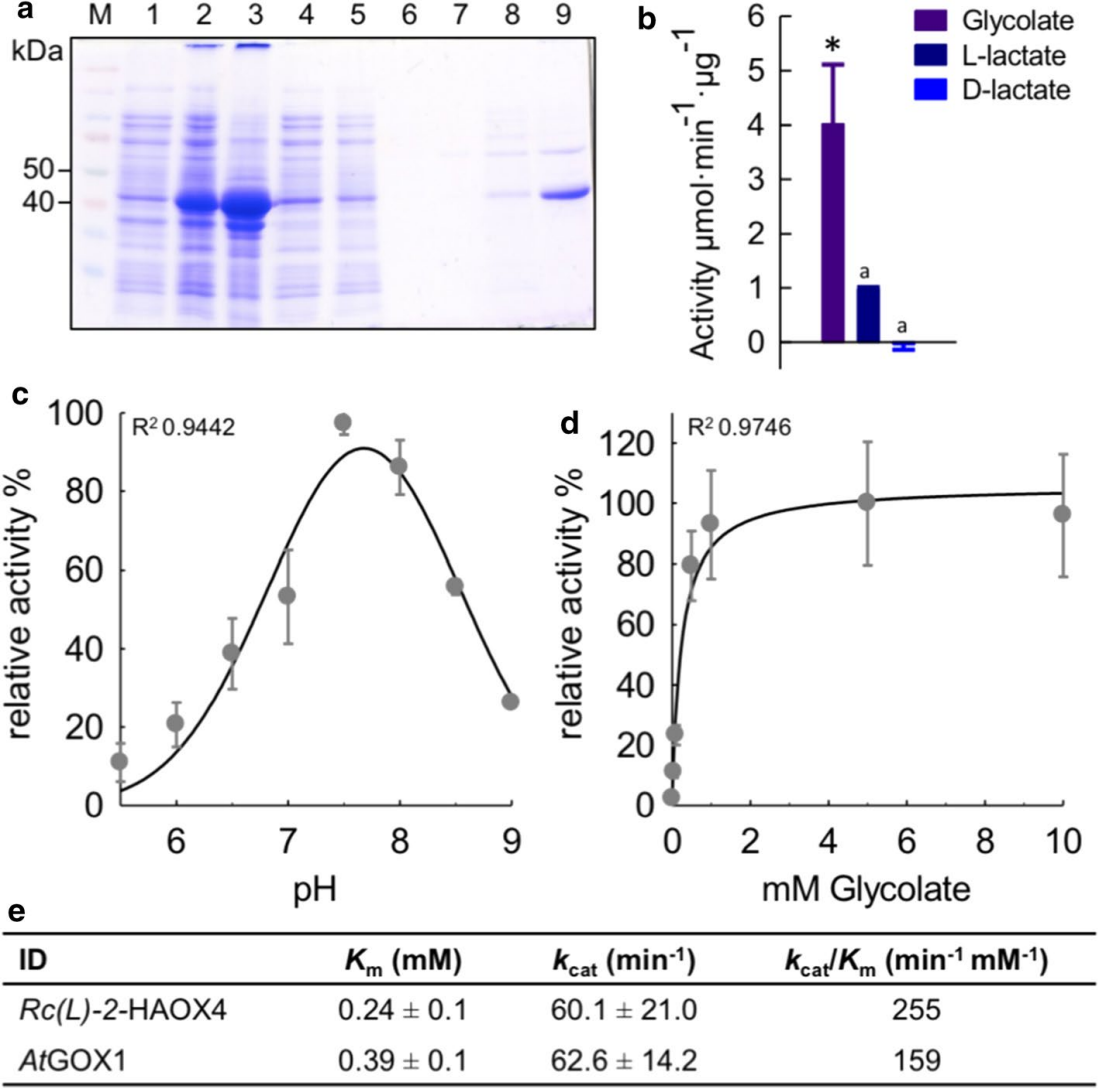
Biochemical properties of Rc(l)-2-HAOX4. **a** Coomassie-stained SDS–polyacrylamide gel of different steps during the isolation of recombinant Rc(L)-2-HAOX4. Crude protein extract of *E. coli* before (1) and after (2) induction of expression with 1 mM IPTG, non-soluble fraction (3) and soluble fraction (4) after cell disruption, flow-through of washing steps (5–7), and eluted recombinant Rc(l)-2-HAOX4 (8 and 9; 44 kDa); *M*   molecular mass standards. **b** Enzymatic activity of recombinant Rc(l)-2-HAOX4 with the possible substrates glycolate, l-lactate, and d-lactate, tested for significant differences with one-way Anova with subsequent Tukey post-hoc test. *Indicate significant difference to all others: *a* significant difference to glycolate. **c** Dependence of Rc(l)-2-HAOX4 activity on the pH in the presence of glycolate as substrate. **d** Saturation kinetic of Rc(l)-2-HAOX4 using glycolate as substrate. **e** Kinetic parameters of Rc(l)-2-HAOX4 and AtGOX1 measured in parallel using glycolate as substrate. *n* = 3, ± SE; *R*^*2*^ coefficient of determination

Rc(l)-2-HAOX4 is highly expressed in leaves (Fig. 2), and phylogenetic analysis (Fig. 1a) indicates that it clusters with the group containing streptophyte GOX; as AtGOX1 and Rc(l)-2-HAOX4 share 90 % aa identity we postulate that Rc(l)-2-HAOX4 represents the (l)-2-HAOX responsible for the detoxification of glycolate formed in the photorespiratory pathway. As this putative GOX is also expressed at high levels in heterotrophic organs, we analyzed the enzymatic properties of the recombinant enzyme. Rc(l)-2-HAOX4 was expressed in *E. coli* and the recombinant protein was isolated by affinity chromatography. The purified protein presented the expected molecular weight of 44 kDa after separation by SDS-PAGE (Fig. 3a).

Activity measurements using different 2-hydroxy acids as substrates at pH 7.5, the pH optimum of all three AtGOX (Engqvist et al. 2015), demonstrate that Rc(l)-2-HAOX4 has the highest activity with glycolate as substrate (100%), followed by l-lactate (25%). In line with the strict stereospecificity of *(l)-2-HAOX* (Maurino and Engqvist 2015), Rc(l)-2-HAOX4 does not use d-lactate (Fig. 3b). In summary, the substrate specificities of Rc(l)-2-HAOX4 are characteristic of an (l)-2-HAOX with GOX activity (Esser et al. 2014; Engqvist et al. 2015; Maurino and Engqvist 2015). The pH-dependent activity profile of Rc(l)-2-HAOX4 determined using glycolate follows a bell-shaped curve with a pH optimum of 7.5 (Fig. 3c). Further kinetic characterization of recombinant Rc(l)-2-HAOX4 conducted with the preferred substrate glycolate at the optimum pH indicated a Michaelis–Menten response (Fig. 3d). Rc(l)-2-HAOX4 exhibits a high affinity for glycolate (*K*_m_ = 0.24 mM) and a high catalytic rate (*k*_cat_ = 60.1 min^−1^), and it thus possesses a high catalytic efficiency (*k*_cat_/*K*_m_ = 255 min^−1^ mM^−1^). These parameters are highly similar to those of the photorespiratory AtGOX1, which we analyzed in parallel (Fig. 3e and Fig. S1).

Our phylogenetic, expressional, and biochemical analyses strongly indicate that the genome of Ricinus possesses a single copy gene encoding a GOX that is found in heterotrophic and autotrophic organs, and has comparable biochemical features to the photorespiratory AtGOX. Thus, in the following we rename Rc(l)-2-HAOX4 to RcGOX.

### The photorespiratory metabolite glycolate is abundant in *Ricinus* heterotrophic organs

**Fig. 4:**
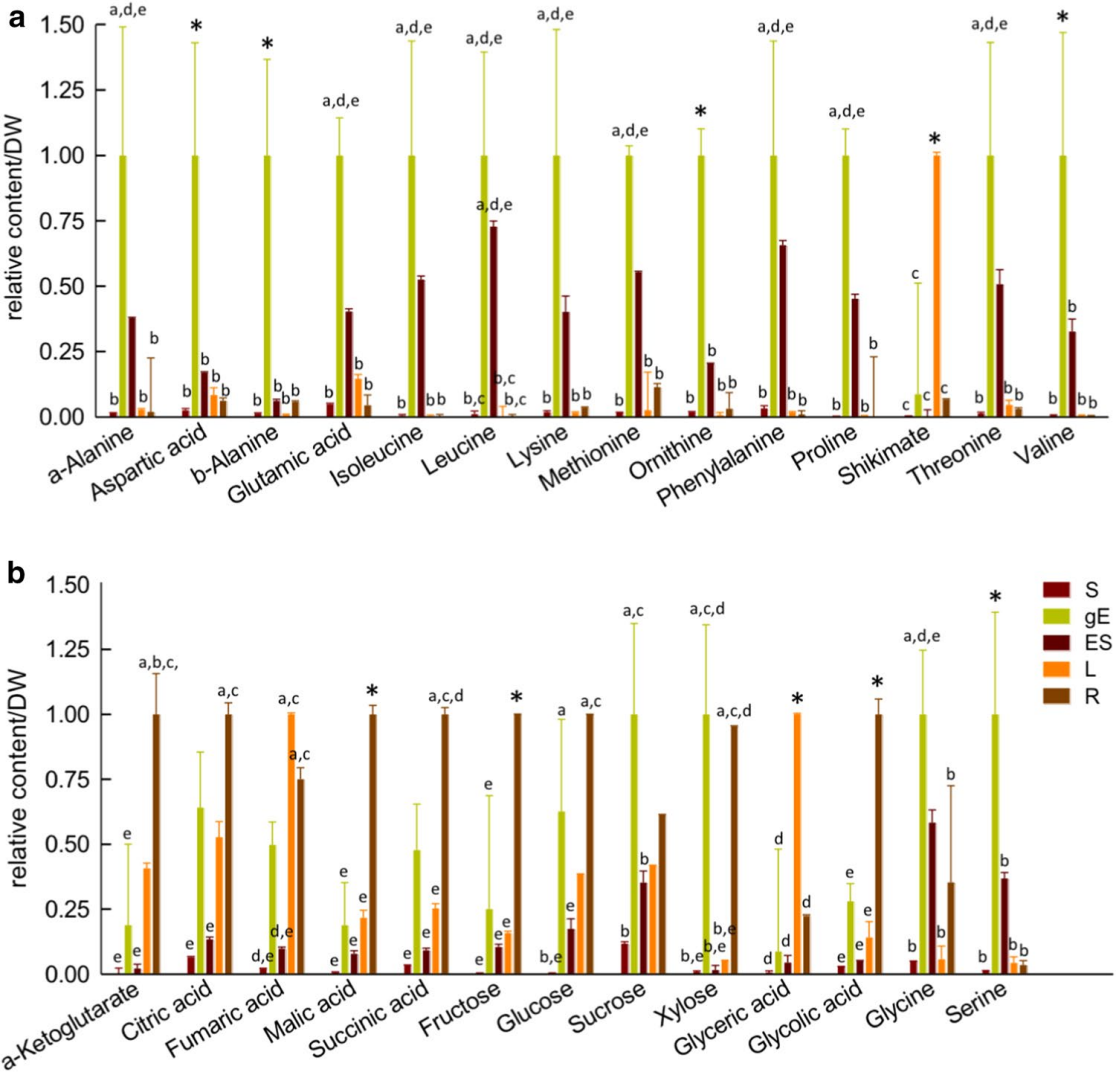
Relative metabolite levels in different organs of *Ricinus communis*. Relative content normalized per dry weight (DW) of amino acids (**a**) and photorespiratory and TCA metabolites and free sugars (**b**) determined by GC/MS. For ease of comparison, the highest relative content measured for each metabolite was set to 1.00 (see Suppl. Table S1 for normalized peak areas). *n* = 3; ± SE; *S* dry seed; *gE* embryo of germinated seed; *ES* endosperm of germinated seed; *L* leaf; *R* root. Values were tested for significant differences with two-way Anova with subsequent Tukey post-hoc test. *Indicate significant difference to all others: *a* = significant difference to *S*, *b* = significant difference to gE, *c* = significant difference to ES, *d* = significant difference to *L* and *e* = significant difference to R

To understand the need for GOX activity in heterotrophic organs of Ricinus, we performed a comparative metabolite profile analysis in leaf, roots, dry seeds, and endosperm and embryo of germinated seeds (Fig. 4 and Table S2). A general profile of amino acids shows that their abundance is highest in the embryonal tissue of germinating seeds, followed by the endosperm of germinating seeds (Fig. 4a); this pattern reflects the high metabolic activity of these fast-growing tissues. The highly abundant amino acids are likely derived from the metabolism of seed storage proteins or through de novo synthesis, as their contents in dry seeds are particularly low (Fig. 4a). Only shikimate, the precursor of the aromatic amino acids, shows a tenfold higher relative amount in leaf than in the heterotrophic organs (Fig. 4a), fitting the fact that the plant shikimate pathway is largely active in chloroplasts (Richards et al. 2006).

We found that relative amounts of TCA cycle intermediates are highest in the root (Fig. 4b), indicating high mitochondrial respiratory activity in this organ. The TCA cycle intermediates show a comparable abundance in leaves and the embryo of germinating seeds, whereas the abundances in dry seeds and the endosperm of germinating seeds are relatively low. The soluble sugars, such as fructose, glucose, sucrose, and xylose show their highest abundances in roots and the embryo of germinating seeds (Fig. 4b).

The photorespiratory metabolites show different profiles in the examined organs (Fig. 4b). Glycerate is predominantly found in the leaf and might derive mainly from photorespiration. As in the case of most amino acids, glycine and serine relative contents are highest in the embryo and endosperm of germinating seeds. Glycolate is predominantly found in roots followed by the embryo of germinating seeds and leaves. Only small amounts of glycolate are present in dry seeds. The presence of glycolate in heterotrophic tissues is not exclusive of Ricinus, as high relative levels of glycolate were also measured in roots of *Arabidopsis thaliana* (Engqvist et al. 2015).

## Discussion

### Different physiological functions of RcGOX in autotrophic and heterotorphic tissues

Our phylogenetic, expressional, and biochemical analyses indicate that the genome of Ricinus possesses a single copy gene, *Rc(l)-2-HAOX4*, encoding a GOX. RcGOX is highly active in autotrophic and heterotrophic organs, where it most likely serves different functions depending on the physiology of the organs.

**Fig. 5:**
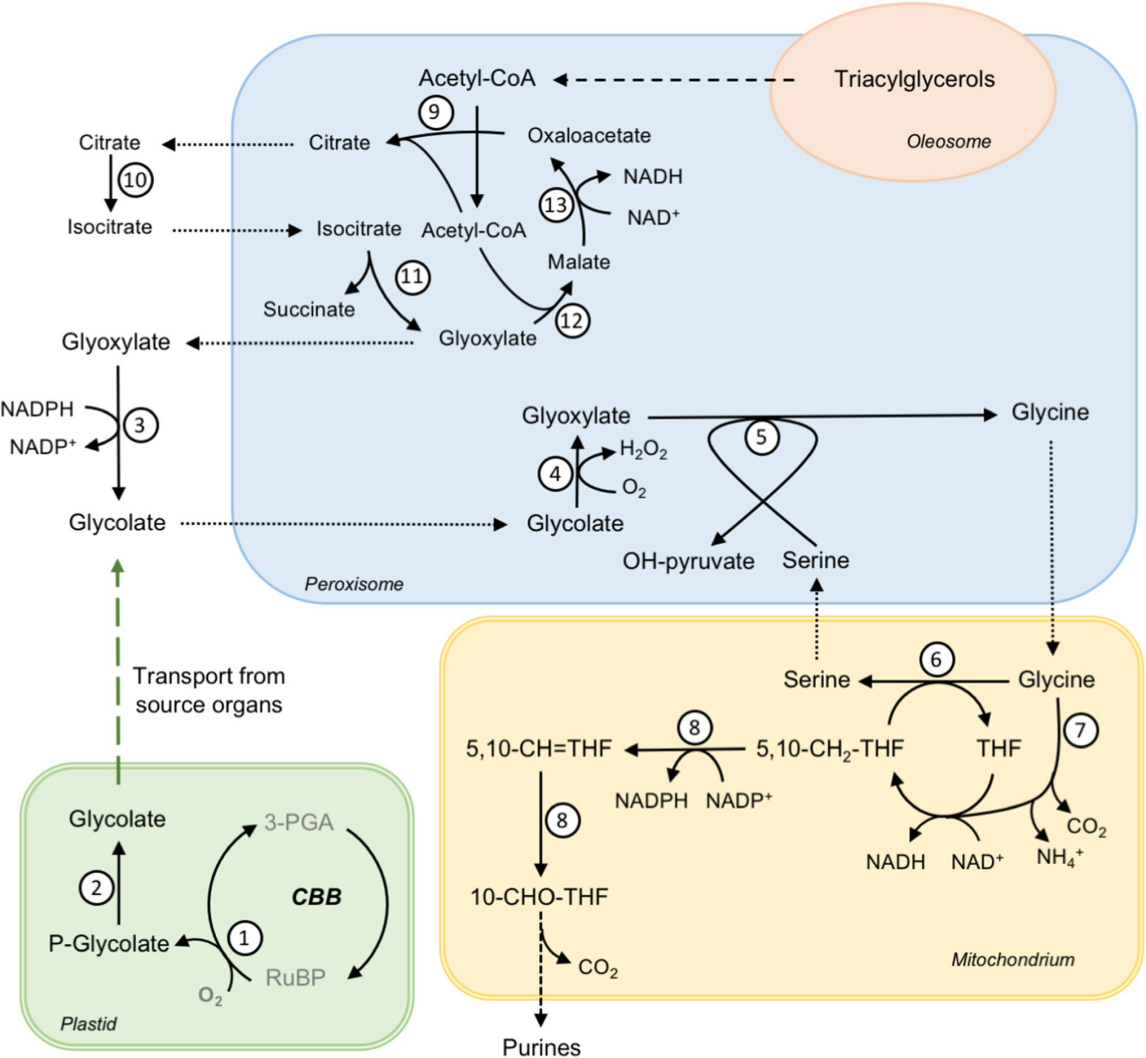
Putative route for respiration of glycolate metabolism to feed mitochondrial demands for NADPH and folates for purine synthesis in fast-growing heterotrophic tissue. 1: Ribulose-1,5-bisphosphat-carboxylase/-oxygenase (RubisCO); 2: phosphoglycolate phosphatase (PGLP); 3: glyoxylate reductase (GLYR); 4: glycolate oxidase (GOX); 5: glutamate:glyoxylate aminotransferase (GGAT)/serine:glyoxylate aminotransferase (SGAT); 6: serine hydroxymethyl transferase (SHMT); 7: glycine decarboxylase complex (GDC); 8: methyltetrahydrofolate dehydrogenase/5,10-methenyltetrahydrofolate cyclohydrolase (MTHF); 9: citrate synthase (CYS); 10: aconitase (ACO); 11: isocitrate lyase (ICL); 12: malate synthase (MSY); 13: malate dehydrogenase (MDH)

In plant metabolism, the known origin and fate of glycolate is found so far within the photorespiratory pathway. In photosynthetic tissues, the sequential action of Rubisco and 2-PG phosphatase (PGLP) is the major pathway for the formation of glycolate in chloroplasts (Fig. 5) and GOX is involved in its detoxification in the peroxisomes (Zelitch et al. 2009). Thus, the function of RcGOX in leaves is the same as that of Arabidopsis GOX1/GOX2: it oxidizes glycolate into glyoxylate as part of the photorespiratory pathway.

In heterotrophic organs RcGOX is most probably involved in the respiration of glycolate for special demands of those tissues. Our results indicate the presence of a substantial amount of glycolate in the embryo during germination, which hints to the production of this 2-hydroxy acid in this non-photosynthetic tissue. Other heterotrophic organs, such as root, may produce or import glycolate from autotrophic sources. Glycolate could be produced through the action of Rubisco and PGLP in heterotrophic tissues, as the small and large subunits of Rubisco are present in those tissues in Arabidopsis (Baerenfaller et al. 2008). Alternatively, glycolate can be produced through the action of cytosolic glyoxylate reductase (Hoover 2007; Simpson et al. 2008; Allan et al. 2009). In this case, glyoxylate would arise through the glyoxylate cycle, which is highly active in germinating oilseeds (Fig. 5) (Eastmond and Graham 2001; Kunze et al. 2006). In this scenario, a fraction of glyoxylate produced in the glyoxylate cycle likely escape to the cytosol, where glyoxylate reductase converts it into glycolate (Fig. 5). Glycolate is most probably transported to the peroxisome and converted back to glyoxylate by the action of GOX (Fig. 5). With this shunt, glyoxylate produced by GOX most probably enter a different peroxisomal metabolon separated from the glyoxylate cycle, which would subsequently enable channeling of glyoxylate to a different downstream part of the metabolic pathway (Fig. 5) (Kunze and Hartig 2013). This theory is strengthened by the fact that the expression of photorespiratory enzymes in heterotrophic tissues as well as the transfer of metabolites across the peroxisomal membrane during the glyoxylate cycle has been already acknowledged (Courtois-Verniquet and Douce 1993; Kunze et al. 2006; Pracharoenwattana et al. 2007; Nunes-Nesi et al. 2014).

### Glycolate respiration most probably fill the demands for mitochondrial NADPH and folate in fast growing heterotrophic organs: a hypothesis

The abundance of glycolate and the presence of a highly active GOX in the embryo and endosperm of germinating seeds and the root of Ricinus indicates an important role of glycolate metabolism in those heterotrophic organs. Feeding experiments using endosperm of Ricinus and radiolabeled glycolate indicated that glycolate is rapidly converted into glyoxylate, glycine, serine, and carbon dioxide (Cossins and Sinha 1967). Glyoxylate directly fed to diverse heterotrophic organs is also rapidly metabolized to serine and glycine (Sinha and Cossins 1965). In agreement with these findings, our metabolite profile indicated large amounts of glycine and serine specifically in the embryo and endosperm of germinating seeds. These amino acids can be produced from glyoxylate through the action of glutamate:glyoxylate aminotransferase/serine:glyoxylate aminotransferase, glycine decarboxylase, and serine hydroxymethyltransferase (Fig. 5).

In addition to amino acid synthesis, the action of the glycine cleavage system is an essential source of one-carbon units (Fig. 5) (Hanson and Roje 2001). In plants, folate metabolism contributes to NADPH production through the conversion of 5,10-methylene-THF to 5,10-methenyl-THF by 5,10-methylene-THF dehydrogenase (Gorelova et al. 2017). In animal cells, serine was also shown to be the major carbon sources for the formation of 10-formyl-THF, the further metabolization of which makes an important contribution to the production of mitochondrial NADPH (Fan et al. 2014). In animal and plant cells, NADPH can be further used in the reduction of glutathione, one of the most important reactive oxygen species (ROS) scavengers (Fan et al. 2014; Gorelova et al. 2017). Controlled levels of ROS are necessary to regulate specific processes during plant cell growth, such as germination (Yazdanpanah et al. 2018), root hair growth (Foreman et al. 2003) and leaf cell expansion (Rodriguez et al. 2002). Thus, folate metabolism can contribute to NADPH production needed for glutathione reduction to maintain ROS levels especially in fast-growing cells. Furthermore, 10-formyl-THF can be used for de novo purine synthesis feeding the demands of nucleotide metabolism in fast-growing embryonic tissue (Hanson and Roje 2001).

In Arabidopsis the phosphorylated pathway of serine biosynthesis is essential to sustain root growth and embryo development (Munoz-Bertomeu et al. 2013; Cascales-Minana et al. 2013). This pathway likely provides the serine involved in folate metabolism as has been demonstrated in mammals (Fan et al. 2014). Nevertheless, it cannot be ruled out that in other plant species there exist a special necessity of strengthening folate metabolism for specific metabolic demands of heterotrophic organs. The results obtained in our work together with existing literature let us propose a hypothesis for a likely function of RcGOX in Ricinus heterotrophic organs (Fig. 5). In this model, glycolate is channeled through RcGOX to fill the NADPH and purine pools. Glycolate is converted by RcGOX into glyoxylate, which is used for the synthesis of serine and glycine. The metabolism of these amino acids likely produces 5,10-methylene-THF, which further transformations produce NADPH and purines (Fig. 5). In summary, we suggest that in heterotrophic organs of Ricinus RcGOX is likely involved in the production of serine to feed the folate pathway for special demands of those tissues; a proposal that deserves further investigations.

#### Author contributions statement

VGM conceived and led the project. VGM and JS designed the work. JS, MH and DM produced data. JS, MH, NL, and VGM contributed to writing the manuscript and generation of the figures.

## Electronic supplementary material

Below is the link to the electronic supplementary material.

Supplementary Fig. S1 Purification and kinetic measurements of AtGOX1. a Coomassie-stained SDS-polyacrylamide gel of different steps during the isolation of recombinant AtGOX1. Crude protein extract of E. coli before (lane 1) and after (lane 2) induction of expression with 1 mM IPTG, non-soluble fraction (lane 3) and soluble fraction (lane 4) after cell disruption, flow-through of washing steps (lanes 5-7), and eluted recombinant AtGOX1 (lanes 8 and 9; 43 kDa); M=Molecular mass standards. b Dependence of AtGOX1 activity on the pH in the presence of glycolate as substrate. c Saturation kinetic of AtGOX1 using glycolate as substrate. R2 = coefficient of determination (DOCX 3151 KB)

Supplementary Table S1 Primers used in 5’-3’ orientation.*, taken from Cagliari et al. (2010) (DOCX 16 KB)

Supplementary Table S2 Normalized peak areas of metabolites normalized per DW and FW measured by GC/MS including results of two-way Anova with subsequent Tukey post-hoc test (XLSX 35 KB)

## Abbreviations

GOX: Glycolate oxidase
(l)-2-HAOX: L-2-hydroxy acid oxidase
lHAOX: Long-chain 2-hydroxy acid oxidases
