# Supplementary figures and images for: The genome of Ricinus communis encodes a single glycolate oxidase with different functions in photosynthetic and heterotrophic organs
Source: Planta. 2020 Nov 10;252(6):100. doi: 10.1007/s00425-020-03504-0 (PMC7655567; doi:10.1007/s00425-020-03504-0)

**Figure S1**


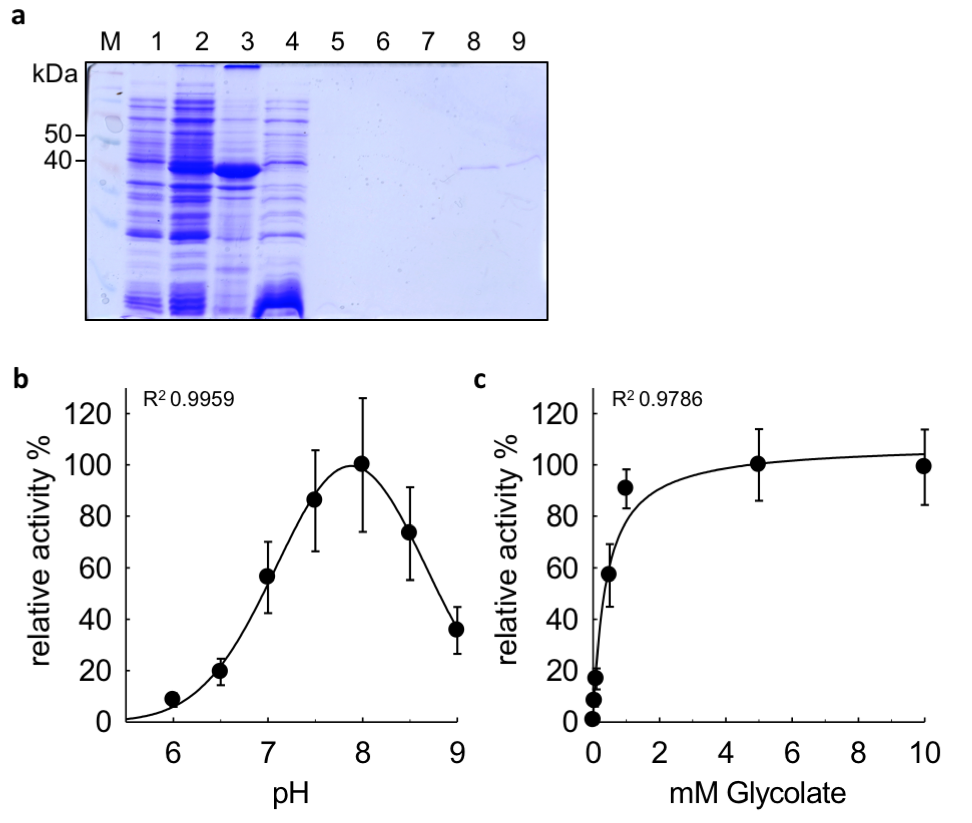

Supplement: Supplementary file 1 — Supplementary Fig. S1 Purification and kinetic measurements of AtGOX1. a Coomassie-stained SDS-polyacrylamide gel of different steps during the isolation of recombinant AtGOX1. Crude protein extract of E. coli before (lane 1) and after (lane 2) induction of expression with 1 mM IPTG, non-soluble fraction (lane 3) and soluble fraction (lane 4) after cell disruption, flow-through of washing steps (lanes 5-7), and eluted recombinant AtGOX1 (lanes 8 and 9; 43 kDa); M=Molecular mass standards. b Dependence of AtGOX1 activity on the pH in the presence of glycolate as substrate. c Saturation kinetic of AtGOX1 using glycolate as substrate. R2 = coefficient of determination (DOCX 3151 KB) [file 425_2020_3504_MOESM1_ESM.docx]
