# Supplementary material for: The genome of Ricinus communis encodes a single glycolate oxidase with different functions in photosynthetic and heterotrophic organs
Source: Planta. 2020 Nov 10;252(6):100. doi: 10.1007/s00425-020-03504-0 (PMC7655567; doi:10.1007/s00425-020-03504-0)
Supplement: Supplementary file 2 — Supplementary Table S1 Primers used in 5’-3’ orientation.*, taken from Cagliari et al. (2010) (DOCX 16 KB) [file 425_2020_3504_MOESM2_ESM.docx]

**Table S1.** Primers used in 5’-3’ orientation.*, taken from Cagliari et al. (2010)

| **Usage** | **Name** | **Sequence 5‘-3‘** |
| --- | --- | --- |
| Heterologous expression | Rc4-pET_F | GGATCCGGAGATAACCAATGTTATGGA |
|  | Rc4-pET_R | GGATCCTTATAACCTGGCAGAGGCA |
| Real-time PCR | Rc1_F | GAATTATTGTCTCCAACCAC |
|  | Rc1_R | TCCTCCAAGACAGAAATAGT |
|  | Rc2_F | CGGCGAGGAACTGATGTATT |
|  | Rc2_R | TCACTGCCAGCCCATAGATT |
|  | Rc3_F | GGAGTACAGAGAGGAACAGA |
|  | Rc3_R | ACGTCTCACTCCATAATCTC |
|  | Rc4_F | GAAGGTGAAGCTGGTATTAGG |
|  | Rc4_R | GGAAGATCCCAGTCAGTTAC |
|  | EF1b_F* | GCAGTTCGGAGCATTGAGAT |
|  | EF1b_R* | GTCGTCCACAATGGTCATCA |
|  | UBC_F | AATCCCGATGACCCATTG |
|  | UBC_R | TTGCTGTTGCCTCGTATTTG |
| Subcelluar localization | FP611_F  FP611_R  Rc4_N-term_fusion_F | GAACACGGGGGACTCTAGAGGATCCATGAATTCACTGATCAAGGAAAATA  ACTAGTCTCGAGAAGACGTCCCAGTTTGGATG  gggacgtcttctcgagactagtATGgagataaccaatgttatg |
|  | Rc4_N-term_fusion_R | Cgatcggggaaattcgagctcttataacagggcgaggaagatc |
|  | Rc4_C-term_fusion_F | AGAACACGGGGGACTCTAGAATGGAGATAACCAATGTTATGGAATATGAG |
|  | Rc4_C-term_fusion_R | cttgatcagtgaattcatcccgggtaacctggcagaggcaac |
